# N, F-Codoped Microporous Carbon Nanofibers as Efficient Metal-Free Electrocatalysts for ORR

**DOI:** 10.1007/s40820-019-0240-x

**Published:** 2019-01-22

**Authors:** Tianle Gong, Ruoyu Qi, Xundao Liu, Hong Li, Yongming Zhang

**Affiliations:** 1 Shanghai Electrochemical Energy Devices Research Center, Shanghai Key Lab of Electrical Insulation and Thermal Aging, School of Chemistry and Chemical Engineering, Shanghai Jiao Tong University, Shanghai, 200240 People’s Republic of China; 2 School of Material Science and Engineering, University of Jinan, Jinan, 250022 People’s Republic of China

**Keywords:** Metal-free catalyst, Oxygen reduction reaction, N, F-codoped, Carbon nanofiber, Polyvinylidene fluoride

## Abstract

Currently, the oxygen reduction reaction (ORR) mainly depends on precious metal platinum (Pt) catalysts. However, Pt-based catalysts have several shortcomings, such as high cost, scarcity, and poor long-term stability. Therefore, development of efficient metal-free electrocatalysts to replace Pt-based electrocatalysts is important. In this study, we successfully prepared nitrogen- and fluorine-codoped microporous carbon nanofibers (N, F-MCFs) via electrospinning polyacrylonitrile/polyvinylidene fluoride/polyvinylpyrrolidone (PAN/PVDF/PVP) tricomponent polymers followed by a hydrothermal process and thermal treatment, which was achieved for the first time in the literature. The results indicated that N, F-MCFs exhibit a high catalytic activity (*E*_onset_: 0.94 V vs. RHE, *E*_1/2_: 0.81 V vs. RHE, and electron transfer number: 4.0) and considerably better stability and methanol tolerance for ORR in alkaline solutions as compared to commercial Pt/carbon (Pt/C, 20 wt%) catalysts. Furthermore, in acidic media, N, F-MCFs showed a four-electron transfer pathway for ORR. This study provides a new strategy for in situ synthesis of N, F-MCFs as highly efficient metal-free electrocatalysts for ORR in fuel cells.

**Electronic supplementary material:**

The online version of this article (10.1007/s40820-019-0240-x) contains supplementary material, which is available to authorized users.

## Highlights

- A new and facile method to synthesize N, F-codoped microporous carbon nanofiber (N, F-MCF) electrocatalysts via electrospinning, hydrothermal process, and thermal treatment.
- Polyvinylidene fluoride is applied as a fluorine source in oxygen reduction reaction (ORR) catalysis for the first time in literature.
- N, F-MCFs exhibit distinguished electrocatalytic activity, stability, and methanol tolerance for ORR in alkaline media.

**Electronic supplementary material:**

The online version of this article (10.1007/s40820-019-0240-x) contains supplementary material, which is available to authorized users.

## Graphical abstract

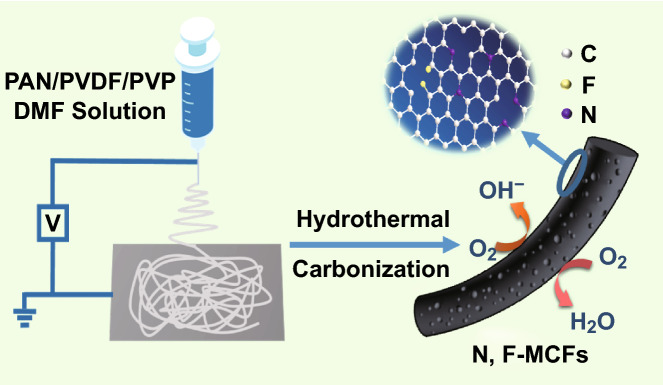

## Introduction

Oxygen reduction reaction (ORR) electrocatalysts for proton exchange membrane fuel cells (PEMFCs) have gained significant attention because of their sluggish kinetic process [1–5]. To date, platinum (Pt)-loaded carbon is considered as the most effective ORR electrocatalyst [6–9]. However, Pt-based catalysts still have several shortcomings, such as poor durability, limited reserves, high cost, and carbon monoxide (CO) poisoning [10, 11]. Currently, metal-free heteroatom-doped carbons are widely considered as promising catalysts to replace Pt-based carbon catalysts in the near future because of their high electrocatalytic activity toward ORR, cost effectiveness, long-term cycling stability, and excellent tolerance to methanol and CO oxidations [12, 13]. Among these materials, nitrogen (N)-doped carbons are extensively studied because the electronegativity of N (3.04) induces charge redistribution of adjacent atoms in an N-doped carbon surface layer, which greatly enhances the ORR activity of carbon electrocatalysts [14–18]. Besides N, other nonmetal atoms with different electronegativities, such as boron (B) [19, 20], sulfur (S) [21, 22], phosphorus (P) [23, 24], and fluorine (F) [25–29], can enhance the ORR activity of carbon catalysts.

In addition, the largest electronegativity is observed in F atoms (4.0). Ishizaki et al. reported that F atoms bonded to ionic and semi-ionic C atoms can act as electron acceptors, which promote charge transfer between the F and C atoms, thereby resulting in higher conductivity and modification of the electronic properties of pristine carbons [30]. Moreover, Lu et al. reported that F doping can improve the wettability of the catalyst surface, thereby facilitating both electrolyte and O_2_ transportation within porous frameworks [31]. Therefore, F doping is advantageous for ORR activities. Moreover, N and F atoms can enhance ORR activities by a synergetic effect. N, F-codoped carbon electrocatalysts, such as carbon black [25], mesoporous carbon [26, 32], graphdiyne [33], porous carbon [34], carbon nanoparticles [30], graphene [35], and graphite nanofibers [27], are widely prepared, and they exhibited excellent properties as ORR electrocatalysts in alkaline media. It is difficult to dope F atoms into carbon matrix; thus, a large number of F sources are required. Currently, NH_4_F is the most commonly used F source. However, the facile decomposition property of NH_4_F increases the synthesis difficulty of F-doped carbons. Further, the F-doped content in obtained carbon samples is less than 1 at%, while the mass amount of NH_4_F used is 20 times more than that of the carbon source [25, 27, 33]. Thus, it is important to develop new F sources and highly efficient F-doping methods.

Besides heteroatom doping, carbon morphology is another key factor that affects catalyst activities. High surface areas along with suitable micropores or mesopores can increase the number of active sites for ORR and facilitate O_2_ transportation during ORR. In this study, we present a facile in situ method to synthesize N, F-codoped microporous carbon nanofibers (N, F-MCFs) as electrocatalysts with high Brunauer–Emmett–Teller (BET) surface area via electrospinning polyacrylonitrile/polyvinylidene fluoride/polyvinylpyrrolidone (PAN/PVDF/PVP) tricomponent polymers followed by a hydrothermal process and thermal treatment. PVDF is used as a source of F and C atoms. PAN acts as a source of N and C atoms. PVP, which is removed by the hydrothermal process, is applied as a porogen for N, F-MCFs. The as-synthesized N, F-MCFs are characterized systematically, and their ORR activities and stabilities are investigated. Benefitted from the N, F-codoped effect and unique nanofiber structure with microporous pore walls, N, F-MCFs exhibit both highly catalytic activity and stabilities for ORR in alkaline solutions. The catalytic activity of N, F-MCFs in acidic solutions is also investigated preliminarily.

## Experimental Methods

### Materials and Chemicals

PAN (*M*_w_ = 150,000 g mol^−1^) and PVP (*M*_w_ = 10,000 g mol^−1^) were purchased from J&K Scientific Ltd. PVDF (Solef 5130) was obtained from Solvay. 5% Nafion^®^ solution (Nafion 117) was obtained from E. I. DuPont Company. Other chemicals, such as *N*,*N*-dimethylformamide (DMF), potassium hydroxide (KOH), and ethanol, were purchased from Sinopharm Chemical Reagent CO., Ltd. and used as received. Deionized water was used throughout the experiments.

### Electrospinning of PAN/PVP/PVDF Membranes

The tricomponent PAN/PVP/PVDF nanofibrous membranes were prepared via facile single-nozzle electrospinning. The PAN (3 wt%), PVP (3 wt%), and PVDF (3 wt%) membranes were mixed in a sealed glass bottle with DMF and magnetically stirred for 24 h at room temperature as a precursor solution. Further, this precursor solution was loaded into a 3-mL plastic syringe connected with a stainless needle of 0.5 mm inner diameter. During the electrospinning process, the operating voltages were 12 kV, the flow rate was 0.2 mL h^−1^, and the collecting distance was 14 cm. The electrospun PAN/PVP/PVDF membranes were peeled off from the aluminum foil.

### Preparation of N, F-MCFs

The electrospun PAN/PVP/PVDF membranes were transferred into a 100-mL Teflon stainless autoclave with deionized water and hydrothermally treated under 110 °C for 6 h to remove PVP. Further, the hydrothermal-treated membranes were washed with deionized water and dried under 100 °C in a blast oven to obtain PAN/PVDF fibrous membranes.

The peroxidation and carbonization of these PAN/PVDF fibers were performed in an electric heating tube furnace. First, the dried PAN/PVDF fibrous membranes were sealed in a graphite boat covered by a carbon paper. Subsequently, the samples were preoxidized in an air atmosphere under 220 °C for 2 h at a heating rate of 2 °C min^−1^. Further, the samples were carbonized in an N atmosphere under 1000 °C for 2 h at a heating rate of 2 °C min^−1^. The as-prepared samples (N, F-MCFs) were cooled down to the room temperature.

### Physical and Electrochemical Characterization

The morphology of N, F-MCFs was characterized by transmission electron microscopy (TEM) and scanning electron microscopy (SEM) using Nova NanoSEM 450 and Talos F200X (both from FEI Company, USA), respectively. X-ray photoelectron spectroscopy (XPS, Kα) analyses were performed on an AXIS UltraDLD X-ray photoelectron spectrometer system equipped with Al radiation as a probe, and the analysis spot size was 400 μm in diameter. Raman spectra were collected by DXR Micro-Raman Spectroscopy (Thermo Fisher Scientific, USA), equipped with a holographic grating of 1800 lines mm^−1^ and a He–Ne laser (532 nm) as the excitation source. BET measurements were performed on an ASAP 2460 surface area and porosimetry analyzer (Micromeritics Instrument Corp., USA).

All electrochemical measurements were performed on an Autolab PGSTAT302 (Metrohm, Netherlands) electrochemical workstation with a standard three-electrode system. A glassy carbon electrode, Ag/AgCl, and Pt wire were used as the working, reference, and counter electrodes, respectively. The catalyst ink consisted of 1-mg sample, 8 μL 5% Nafion 117 solution, ethanol, and water suspension. After ultrasonic homogenization, the ink was coated on a glassy carbon electrode (working electrode), which led to a catalyst loading of about 0.3 mg cm^−2^ for all working electrodes. Linear sweep voltammetry (LSV) measurement was performed by glassy carbon rotating disk electrode (RDE) cathodically scanned with varying rotating speed from 400 to 2000 rpm at a rate of 5 mV s^−1^. The electron transfer number per O_2_ during the ORR process was calculated by the LSV curves and Koutecky–Levich (K–L) equations (Eqs. 1–3)

1 $$\frac{1}{J} = \frac{1}{{J_{\text{L}} }} + \frac{1}{{J_{\text{K}} }} = \frac{1}{{B\omega^{1/2} }} + \frac{1}{{J_{\text{K}} }}$$

2 $$B = 0.62nFD_{{{\text{O}}_{ 2} }}^{2/3} C_{{{\text{O}}_{ 2} }}^{\text{b}} v^{ - 1/6}$$

3 $$\left| {J_{\text{k}} } \right| = nFk_{\text{f}} C_{{{\text{O}}_{ 2} }}^{\text{b}}$$

where *J* is the measured current density, *J*_K_ and *J*_L_ are the kinetic and diffusion-limiting current densities, respectively, $\omega$ is the angular velocity (rad s^−1^), *F* is the Faraday constant (96,485 C mol^−1^), *n* is the transfer electron number, $C_{{{\text{O}}_{ 2} }}^{\text{b}}$ is the bulk concentration of O_2_ (1.2 × 10^−3^ mol cm^−3^), $D_{{{\text{O}}_{ 2} }}^{2/3}$ is the diffusion coefficient of O_2_ in the electrolyte (1.9 × 10^−5^), *v* is the kinematic viscosity of the electrolyte (0.01 cm^2^ s^−1^), and *k*_f_ is the electron transfer rate constant.

## Results and Discussion

### Structure and Morphology of N, F-MCFs

**Scheme 1:**
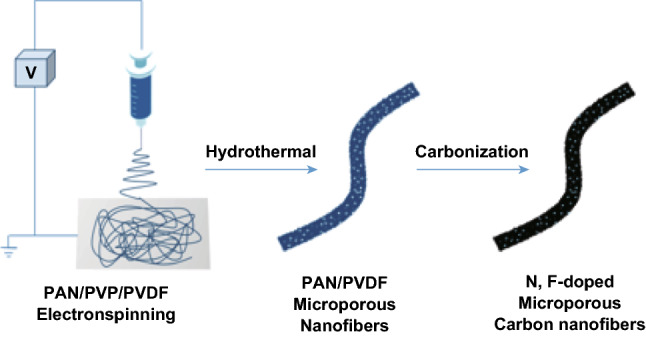
Schematic illustration of N, F-MCFs ORR catalysts

The overall preparation process for N, F-MCFs is illustrated in Scheme 1. As shown in Scheme 1, the PAN/PVP/PVDF tricomponent nanofibers are first obtained by electrospinning a mixture of PAN, PVP, and PVDF in the DMF solution. Further, they are hydrothermally treated under 110 °C for 6 h, and then preoxidized and carbonized at 220 and 1000 °C, respectively, thereby resulting in N, F-MCFs. There are two mass ratios of PAN, PVP, and PVDF for the prepared N, F-MCFs, 1/1/1 and 1/1/1.5, which are separately named as N, F-MCFs-A and N, F-MCFs-B, respectively. To investigate the effects of pores and F atoms on N, F-MCFs, we synthesized N-MCFs-C without F atom doping from PAN/PVP bicomponent polymers and N, F-CFs-D without micropores from PAN/PVDF bicomponent polymers for comparison.

**Fig. 1:**
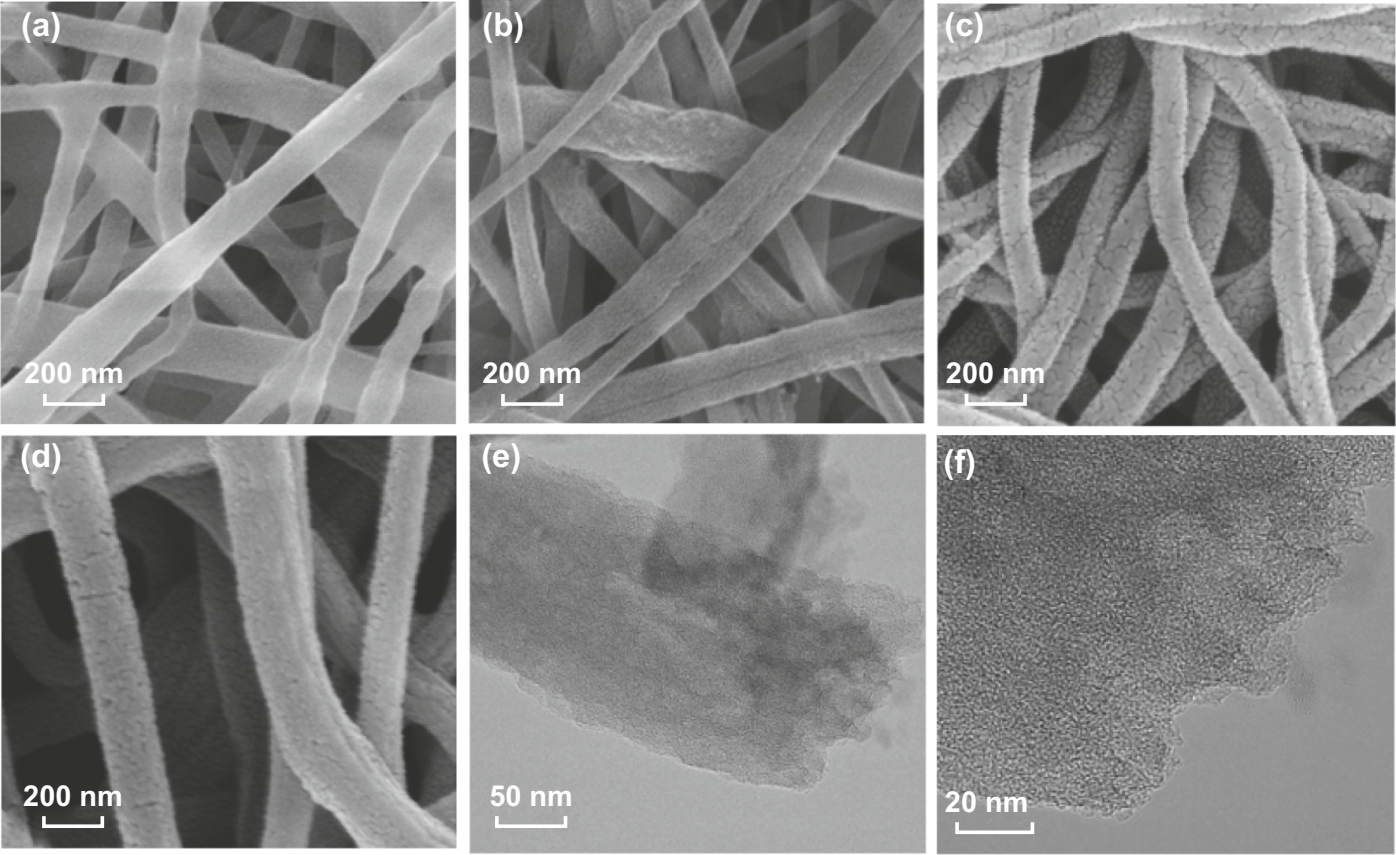
SEM images of **a** electrospun PAN/PVP/PVDF nanofibers, **b** PAN/PVDF porous nanofibers after hydrothermal treatment, N, F-MCFs with different compositions, **c** PAN/PVP/PVDF = 1/1/1, and **d** PAN/PVP/PVDF = 1/1/1.5. **e**, **f** TEM images of N, F-MCFs

The morphology of electrospun PAN/PVP/PVDF nanofibers for the entire preparation process was characterized by SEM. As shown in Fig. 1a, smooth and uniform electrospun PAN/PVP/PVDF nanofibers exhibited a mean diameter of about 180 nm with random orientation. After removing PVP of the hybrid fibers by hydrothermal treatment, the surface of the nanofibers becomes rough and uneven, as shown in Fig. 1b. We can observe that some pores appear on the fibers. However, the continuous and uniform fiber structure is maintained after the hydrothermal treatment as well. The heat treatment of the porous nanofibers includes two processes, pre-oxidation and carbonization. During pre-oxidation in air at 220 °C, PAN undergoes cyclization and partial dehydrogenation, which make fibers more stable during subsequent high-temperature carbonization and create more defects for doping heterogeneous atoms [36]. As shown in Fig. 1c, d, the obtained N, F-MCFs became thinner and denser after carbonization, and cracks formed on their surface. The nanofiber structure with pores and the amorphous carbon microcrystalline structure can be observed in the TEM images of N, F-MCFs-A (Fig. 1e, f).

**Fig. 2:**
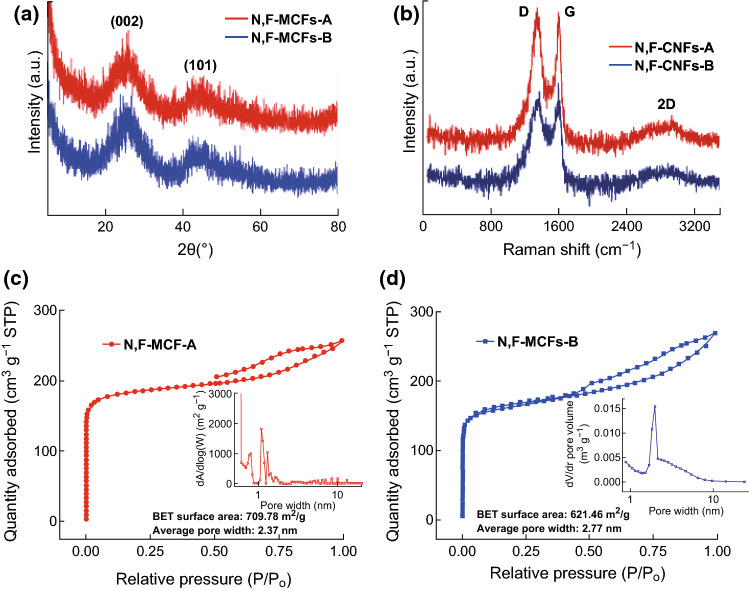
**a** XRD patterns, **b** Raman spectra, **c**, **d** N_2_ adsorption isotherms (inset of **c** and **d** is pore size distributions of N, F-MCFs-A and N, F-MCFs-B)

The crystal structure and degree of graphitization are further characterized by XRD analyses and Raman spectrum. Two broad peaks at around 2*θ* = 25° and 44°, corresponding to the (002) and (100) planes of carbon, respectively, validate the amorphous carbon structure in Fig. 2a. Figure 2b shows three typical D, G, and 2D bands at about 1344, 1598, and 2798 cm^−1^, respectively. The D-band represents the defects and disordered structure of carbon lattice, while the G-band is a characteristic feature of in-plane vibration of *sp*^2^ bonded carbon atoms, which indicates the ordered structure of the carbon materials. It is known that the ratio of D-band and G-band (*I*_D_/*I*_G_) is attributed to determine the degree of graphitization or the defect density of carbon materials. The *I*_D_/*I*_G_ ratios of N, F-MCFs-A and N, F-MCFs-B are as high as 2.98 and 2.31, respectively, which suggests that many defect sites and disordered structures are caused by doping N and F atoms. Moreover, they are in accordance with the broad peak of (002) obtained from the XRD results.

The pore structure of N, F-MCFs was investigated by adsorption–desorption isotherms of N_2_ at − 196 °C. As shown in Fig. 2c, d, both isotherms are between type I isotherms with type H4 hysteresis loops following IUPAC classification, thereby indicating the existence of micropores. The BET surface areas of N, F-MCFs-A and N, F-MCFs-B are 709.78 and 621.46 m^2^ g^−1^, respectively. The corresponding pore size distributions of the two samples are calculated by Barrett–Joyner–Halenda (BJH) desorption, and their average pore widths are 2.37 and 2.77 nm. The large BET surface area and the existence of micropores are advantageous for high ORR activities.

**Fig. 3:**
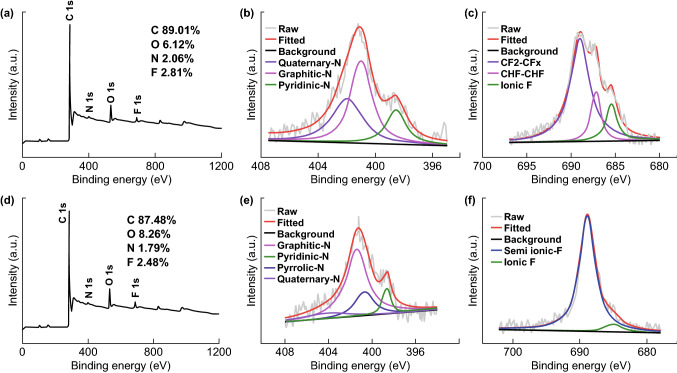
**a**, **d** XPS spectra of N, F-MCFs-A and B. High-resolution XPS spectra of **b**, **e** N 1s and **c**, **f** F 1s of N, F-MCFs-A and B

The elemental composition and the contents of N and F heteroatoms onto the catalysts surface were characterized by XPS measurements. The XPS survey spectra for N, F-MCFs-A and N, F-MCFs-B showed the existence of the C 1s, O 1s, N 1s, and F 1s peaks. The XPS quantitative result demonstrated that the relative surface mass ratios of C, O, N, and F are 89.01, 6.12, 2.06, and 2.81% in N, F-MCFs-A, and 87.48, 8.26, 1.79, and 2.48% in N, F-MCFs-B, respectively. The high-resolution N 1s spectrum can be further deconvoluted into four peaks centered at 398.5 ± 0.2 eV, 400.1 ± 0.2 eV, 401.1 ± 0.2 eV, and 404 ± 0.2 eV, corresponding to pyridinic N, pyrrolic N, graphitic N, and quaternary N, respectively. The relative content of pyridinic N and graphitic N is much higher than that of pyrrolic N and quaternary N, which may contribute to the high ORR activity of the catalyst [26, 27, 34]. The method of doping F atoms via PVDF can obtain higher content (> 2%) than those methods discussed in the earlier literature, which could be beneficial to the ORR electrocatalytic activity. The high-resolution F 1s spectrum was usually deconvoluted into semi-ionic F (688.8 ± 0.2 eV) and ionic F (685.4 ± 0.2 eV). These two peaks can be observed in the F 1s spectrum of N, F-MCFs-B in Fig. 3f, while the F 1s spectrum of N, F-MCFs-A can be deconvoluted into three peaks in Fig. 3e, which include ionic F (685.4 ± 0.2 eV) and two kinds of semi-ionic F, CH_2_–CF_2_ (689 ± 0.2 eV) and CHF–CHF (687.2 ± 0.2 eV). We compared the high-resolution F 1s spectrum of F-monodoped catalyst (prepared by PVDF carbon fibers catalysts) with N, F-MCFs in Fig. S4. The binding energy of the semi-ionic F in F-doped catalysts is lower than that in N, F-codoped catalysts, and the ionic F content of F-monodoped catalyst is much lower than N, F-codoped catalysts, which are probably because of the synergistic interactions between the F and N atoms. It is observed that ionic F can result in higher electrical conductivity and modification of electronic structures of carbon frameworks. Compared to the mass ratio of ionic F in N, F-MCFs-B (7.18%), the higher mass ratio of the ionic F in N, F-MCFs-A (14.83%) can provide more active sites for ORR to enhance the activity of the catalysts.

### Electrocatalytic Activities Toward ORR of N, F-MCFs

**Fig. 4:**
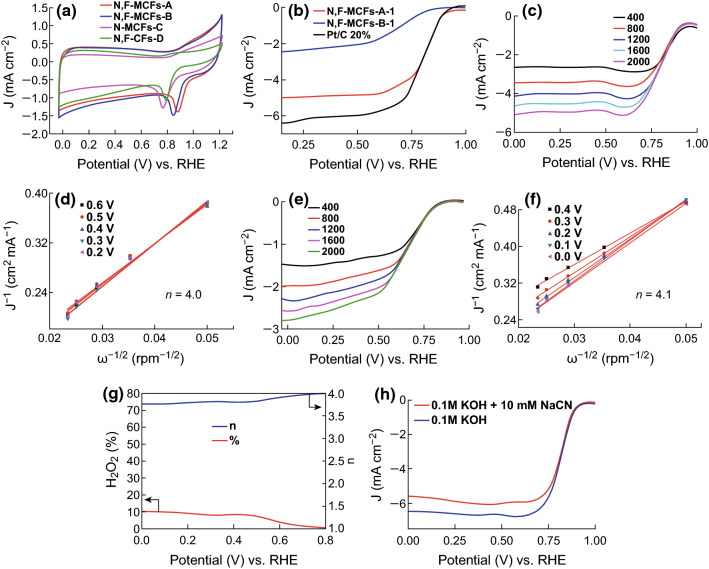
**a** CV curves of N, F-MCFs-A; N, F-MCFs-B; N-MCFs-C; and N, F-CFs-D in O_2_-saturated 0.1 M KOH with a scan rate of 10 mV s^−1^. **b** LSV curves of N, F-MCFs-A; N, F-MCFs-B; and commercial 20% Pt/C in O_2_-saturated 0.1 M KOH with a scan rate of 10 mV s^−1^. LSV curves and K–L plots of **c**, **d** N, F-MCFs-A and **e, f** N, F-MCFs-B. **g** Percentage of H_2_O_2_ yield and electron transfer number (*n*) of N, F-MCFs-A. **h** LSV curves of N, F-MCFs-A with and without 10 mM NaCN in O_2_-saturated 0.1 M KOH with a scan rate of 10 mV s^−1^

The electrocatalytic activities of N, F-MCFs were first evaluated by cyclic voltammetry (CV) measurements in N_2_- and O_2_-saturated 0.1 M KOH solution. The CV curves of N, F-MCFs-A and N, F-MCFs-B in N_2_- and O_2_-saturated 0.1 M KOH solution are shown in Fig. S1. Figure 4a shows that the CV curves of N, F-MCFs-A and N, F-MCFs-B present two peak potentials at 0.881 and 0.846 V, respectively, which are higher than those of N-MCFs-C (prepared from PAN/PVP bicomponent polymers, without F-doped atoms) and N, F-CFs-D (prepared from PAN/PVDF bicomponent polymers without micropores). The more positive peak potentials indicate that more active sites for ORR are created by the high BET surface area and synergistic effect of the codoped heteroatoms. As shown in Fig. S2, the onset potential, half-wave potential, and limiting current density of N, F-MCFs-A are all higher than those of N-MCFs-C, which can also prove that F doping improves the ORR activity. To further investigate the high ORR catalytic activity of N, F-MCFs-A and N, F-MCFs-B, the linear sweep voltammetry (LSV) measurements were performed via a rotating disk electrode (RDE) in O_2_-saturated 0.1 M KOH solution. As depicted in Fig. 4b, N, F-MCFs-A present more positive onset potential (0.94 V vs. RHE) than that of N, F-MCFs-B (0.87 V vs. RHE) and it is also more approaching to that of the commercial Pt/C (0.95 V vs. RHE) because of its larger BET surface area and more doped content of N and F. The high ORR electrocatalytic activity of N, F-MCFs-A can also be gleaned from its higher half-wave potential (0.81 V vs. RHE) than that of N, F-MCFs-B (0.71 V vs. RHE) and close to commercial Pt/C (JM20, 0.83 V vs. RHE). However, N, F-MCFs-A exhibit a lower limiting current density (4.9 mA cm^−2^) than Pt/C (6.1 mA cm^−2^). In addition, because of concerning about the impact of glass corrosion [37], we measured the LSV curves of N, F-MCFs-A and Pt/C in O_2_-saturated 0.1 M KOH solution with Teflon container (Fig. S5) and confirmed that in the short test time poison of Pt/C catalyst by impurities released from the glass cell in alkaline medium should be very little and not detectable.

To further estimate the ORR reaction kinetics of N, F-MCFs, a series of LSV tests were carried out with various rotation speeds from 400 to 2000 rpm in an O_2_-saturated 0.1 M KOH electrolyte (Fig. 4c, e). On the basis of LSV curves at different rotations, the Koutecky–Levich plots and the electron transfer numbers (*n*) for ORR were obtained from the K–L equations. The great linearity and parallelism of the K–L plots (Fig. 4d, f) suggest a direct four-electron pathway for better ORR efficiency. The average *n* value of N, F-MCFs-A and N, F-MCFs-B is 4.0 and 4.1, which suggested that the complete reduction of O_2_ to OH^−^ over N, F-MCFs by a four-electron transfer process in 0.1 M KOH. For further confirmation of 4e^−^ selectivity, the yield of H_2_O_2_ was measured via rotating ring-disk electrode (RRDE) in Fig. S3. The percentage of H_2_O_2_ of N, F-MCFs-A is below 10%, and *n* is about 3.75 in the potential range from 0 to 0.8 V versus RHE (Fig. 4g) in 0.1 M KOH, which indicates the low peroxide formation and promising ORR activity. The poisoning experiment with CN^−^, which can strongly bond to active metal sites, was performed to identify the active sites of the prepared catalysts [38]. As shown in Fig. 4h, although the limiting current density drops a little, the onset potential and half-wave potential of N, F-MCFs-A almost unchanged with and without CN^−^ in 0.1 M KOH. That means the catalytic active sites in N, F-MCFs-A are primarily derived from the F-doped and N-doped carbon sites rather than from other metallic coordination sites.

**Fig. 5:**
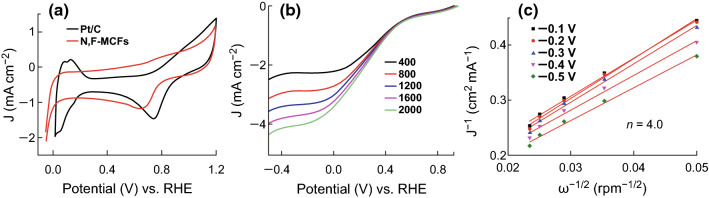
**a** CV curves of N, F-MCFs-A and Pt/C 20% in O_2_-saturated 0.5 M H_2_SO_4_ with a scan rate of 10 mV s^−1^. **b** LSV curves and **c** K*–*L plots of N, F-MCFs-A in O_2_-saturated 0.5 M H_2_SO_4_ with a scan rate of 10 mV s^−1^

The ORR electrocatalytic performance of N, F-MCFs was also tested in acid media. In Fig. 5a, CV measurements in O_2_-saturated 0.5 M H_2_SO_4_ show that the curve peak of N, F-MCFs-A is at 0.649 V (vs. RHE), at which the potential is negative as compared to that of commercial Pt/C (0.748 V vs. RHE). Similarly, LSV measurements (Fig. 5b) in O_2_-saturated 0.5 M H_2_SO_4_ with various rotating speeds from 400 to 2000 rpm and a scan rate of 10 mV s^−1^ also exhibit slightly poor onset potential (0.635 V vs. RHE) and half-wave potential (0.257 V vs. RHE). However, the electron transfer number is calculated to be approximately 4.0 for N, F-MCFs in 0.5 M H_2_SO_4_ from the corresponding K–L plots (Fig. 5c), which indicates that N, F-MCFs have a probable application prospect for ORR electrocatalytic activity in acid media.

**Fig. 6:**
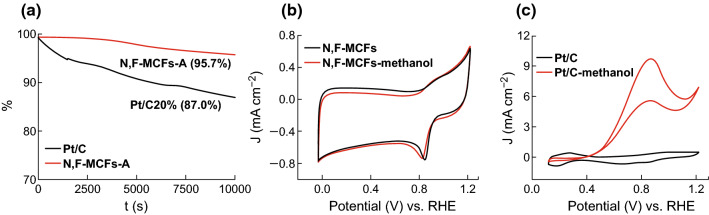
**a** Current–time (*i*–*t*) chronoamperometric response of N, F-MCFs-A and Pt/C 20% in O_2_-saturated 0.1 M KOH at a rotating rate of 1600 rpm for 10,000 s. **b** CV curves of N, F-MCFs-A and **c** Pt/C 20% with and without 3 wt% CH_3_OH in 0.1 M KOH

Furthermore, the durability and tolerance to methanol oxidation of N, F-MCFs-A in 0.1 M KOH were also investigated. The current–time (*i*–*t*) chronoamperometric responses of N, F-MCFs-A and commercial Pt/C were measured under 0.6 V (vs. RHE) in O_2_-saturated 0.1 M KOH at a rotating rate of 1600 rpm for 10,000 s. As shown in Fig. 6a, the relative current density of N, F-MCFs-A decreased more slowly than commercial Pt/C with continuous reaction. It retained a superior higher relative current of 95.7%, while commercial Pt/C only retained 87.0%. The CV curves of methanol tolerance test (Fig. 6b, c) showed that methanol has almost no effect on N, F-MCFs-A; however, a typical methanol oxidation/reduction curve can be observed for Pt/C. Thus, N, F-MCFs-A exhibit not only higher stability but also better methanol resistance than commercial Pt/C, which indicates that N, F-MCFs-A can be a practical metal-free ORR catalyst in fuel cells.

## Conclusions

In summary, we demonstrated a facile method to synthesize N, F-MCF electrocatalysts via electrospinning PAN/PVDF/PVP tricomponent polymers followed by the hydrothermal process and thermal carbonization. N, F-MCFs exhibited distinguished electrocatalytic activity for ORR in alkaline media, including higher onset potential (0.94 V vs. RHE), half-wave potential (0.81 V vs. RHE), and electron transfer number (4.0) because of their unique nanofiber structure with microporous pore walls and synergistic effect of high doped F and N content. In acidic media, the N, F-MCFs also exhibited a four-electron transfer pathway for ORR. In addition, the N, F-MCFs showed outstanding tolerance to methanol and superior stability (95.7%) compared to commercial Pt/C catalysts. As a result of all the superior electrocatalytic performance, this work provides an efficient pathway of in situ synthesis of N, F-MCFs as a highly active metal-free ORR electrocatalyst in the further application of fuel cells.

## Electronic supplementary material

Below is the link to the electronic supplementary material.

Supplementary material 1 (PDF 372 kb)
