# Supplementary material for: N, F-Codoped Microporous Carbon Nanofibers as Efficient Metal-Free Electrocatalysts for ORR
Source: Nanomicro Lett. 2019 Jan 22;11:9. doi: 10.1007/s40820-019-0240-x (PMC7770828; doi:10.1007/s40820-019-0240-x)
Supplement: Supplementary file 1 — Supplementary material 1 (PDF 372 kb) [file 40820_2019_240_MOESM1_ESM.pdf]

Supporting Information for

N, F-Codoped Microporous Carbon Nanofibers as Efficient Metal-Free Electrocatalysts for ORR

Tianle Gong[1], Ruoyu Qi[1], Xundao Liu[2], Hong Li[1,]*, Yongming Zhang[1]

[1]Shanghai Electrochemical Energy Devices Research Center, Shanghai Key Lab of Electrical Insulation and Thermal Aging, School of Chemistry and Chemical Engineering, Shanghai Jiao Tong University, Shanghai 200240, People's Republic of China

[2]School of Material Science and Engineering, University of Jinan, Jinan 250022, People's Republic of China

*Corresponding author. E-mail: lh102@sjtu.edu.cn (Hong Li)

Supplementary Figures

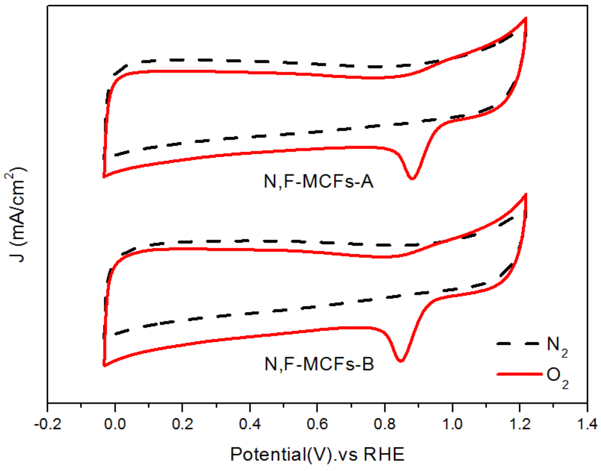

Fig. S1 CV curves of N, F-MCFs-A and N, F-MCFs-B in N_2-saturated and O_2-saturated 0.1 M KOH with a scan rate of 10 mV s^{-1}

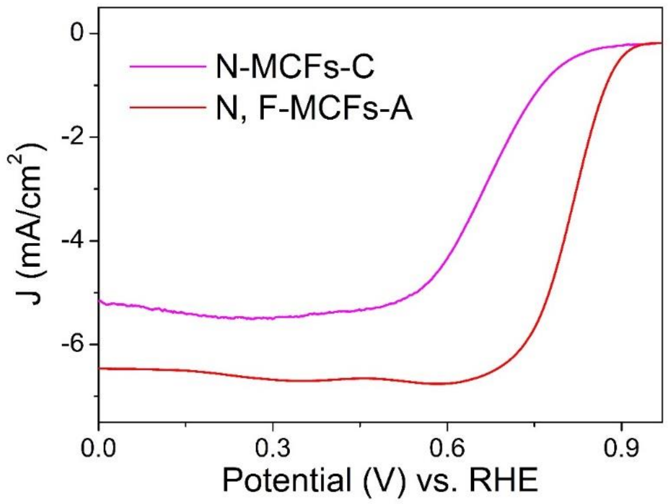

Fig. S2 LSV curves of N, F-MCFs-A and N-MCFs-C in O_2-saturated 0.1 M KOH with a scan rate of 10 mV s^{-1}

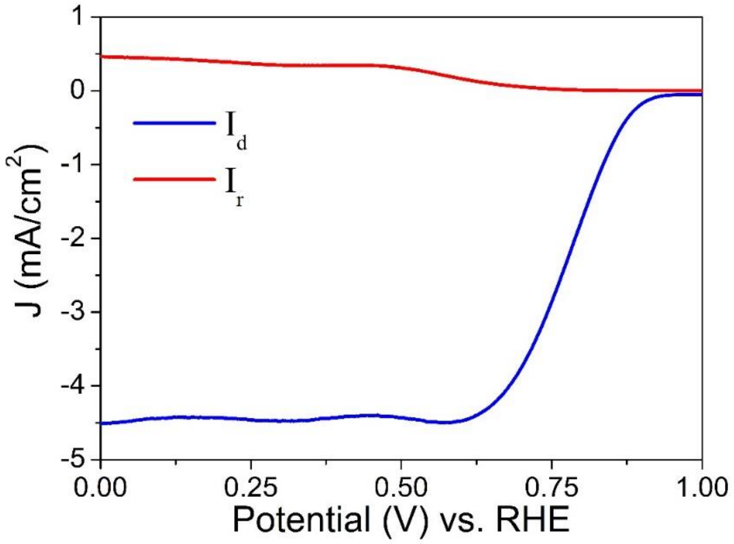

Fig. S3 LSV curves of N, F-MCFs-A via RRDE in O_2-saturated 0.1 M KOH with a scan rate of 10 mV s^{-1}

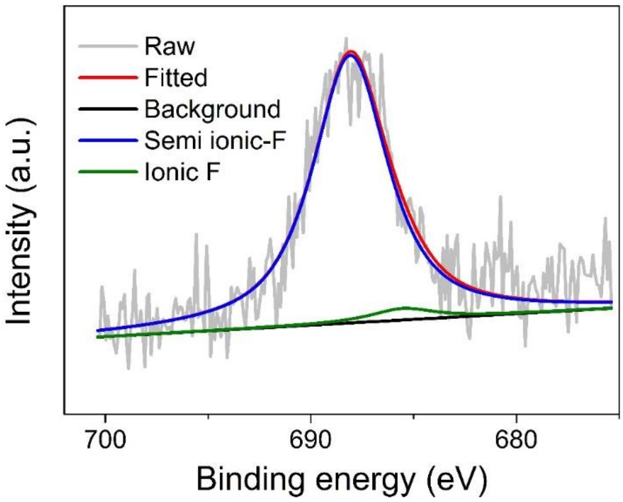

Fig. S4 High-resolution XPS spectra of and F1s of F-monodoped catalysts

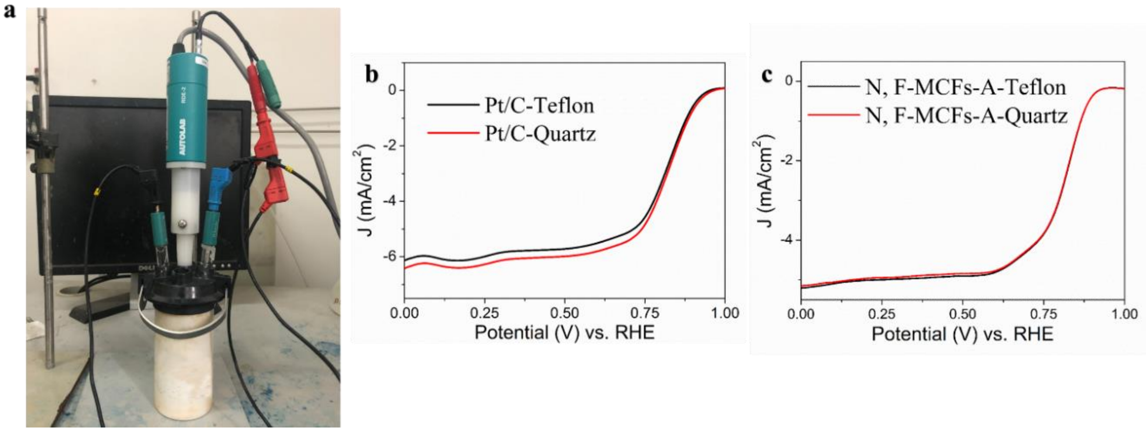

Fig. S5 a Photo of electrochemical cell with Teflon container. **b** LSV curves of Pt/C 20% in Teflon container and quartz cell with O_2-saturated 0.1M KOH at 1600 rpm. **c** LSV curves of N, F-MCFs-A in Teflon container and quartz cell with O_2-saturated 0.1M KOH at 1600 rpm
